# The wicked problem of B(A)ME degree award gaps and systemic racism in our universities

**DOI:** 10.3389/fsoc.2022.971923

**Published:** 2022-10-04

**Authors:** Iwi Ugiagbe-Green, Freya Ernsting

**Affiliations:** Department of Accounting, Finance and Banking, Manchester Metropolitan University Business School, Manchester, United Kingdom

**Keywords:** degree award gap, race, wicked problem, intersectionality, higher education, critical race theory

## Abstract

The independent regulator for higher education in England, the Office for Students (OfS), set new national targets in late 2018 to achieve equality of opportunity in higher education by tackling degree award gaps. The sector response to this was to measure degree award gaps between B(A)ME students and white students in their higher education institutions. Analysis of degree award gaps using quantitative methods has revealed an “unexplained gap”. We argue that the existence of this “unexplained gap” is evidence of “systemic racism”. However, the factors influencing a degree award and their associated gaps across different racialized groups of students are so complex, that its problematisation, never-mind its solution is inherently complex. It is our view, therefore, racialized degree award gaps are a wicked problem. Despite this, it is also our view that it is an important social justice endeavor that we must still seek to address as a sector. To do so, we propose a mixed methods approach that uses dynamic centring and an intersectional lens to better understand the experiences of racialized students within the higher education “system”. Current quantitative analysis of degree award gaps simply tells us how different groups of racialized students experience the system. In using a mixed methods approach in the way we outline, we may better understand the racialized lived experience of our students and the factors influencing the experience of different racialized groups within the “system”. This solution-focused approach can help create opportunities that enable students to better navigate social structures and systems and improve their experience in the system. However, this will not address the wicked problem of degree award gaps itself, which is complex, pervasive, and messy.

## Introduction

A wicked problem has no determinable stopping point due to its complexity in nature. Coined by Rittel and Webber (1973), within their social policy research, they explain that a wicked problem is difficult or impossible to solve. They suggest the difficulty results from contradictory, incomplete, and changing requirements, that can be challenging to identify in the first place. In this paper, we propose that racialized degree awarding gaps are a wicked problem (Conklin, 2005, 2009). The rationale for framing degree award gaps as a wicked problem is that at the root of degree award gaps are complex issues anchored in systemic racism. These issues are persistent, pervasive, and have existed for at least 25 years (Codiroli-Mcmaster, 2021). We propose that the quantitative measurement approaches adopted by UK higher education institutions (HEIs) to try and solve these complex issues are limited. We suggest that although degree award gaps cannot be solved, we can better understand and address the gaps through the adoption of a more holistic mixed methods measurement and intersectional analysis.

## Background—Racialized degree award gaps

The higher education sector in England uses the problematic ethnic terms, BAME (Black, Asian, and Minority Ethnic) or Black and Minority Ethnic (BME) to categorize into one homogeneous grouping anyone who is not racialized as white. One fundamental issue with this is that ethnicity is different from race. Ford and Kelly (2005) explain that ethnicity is mostly used as a social-political construct, and includes shared origin, shared language, and shared cultural traditions; whilst race is a complex, multidimensional social construct. The category of “white” is not a category of ethnicity, but a category of race. However, “BAME” and “BME” are terms of ethnicity used as the language of the higher education sector to make comparisons between white and non-white individuals. As such, ethnicity is used as a proxy for race. This framing is hugely problematic for lots of reasons outside of the scope of discussion for this paper. That said, as this is the language of the sector, we use the terms “BAME” and “BME” when citing reports and data sources relating to degree awarding gaps, and an alternative one when we are framing students not racially categorized as white.

Our own terminology in describing groups of students not racially categorized as white is the term “racially minoritized”. This term “racially minoritized”, acknowledges that race is a socially constructed categorization of people based on physical traits, like skin color. Despite non-white people making up the global majority, it is non-white people who are homogenized into categories of BAME and BME for analysis and therefore, minoritized. This practice of homogenization is an approach that also centers on being white as the norm and makes comparisons of analysis on that basis.

Our discussion now moves from problematic issues associated with racial literacy to that of degree award gaps. Measurement of the degree award gaps within the UK higher education sector is currently led by organizations such as Advance HE, which publishes award gap statistical reports, and the sector's independent regulator, the Office for Students (OfS), which publishes information on degree awarding gaps. Advance HE is the leading UK higher education sector agency. It was formed from the merger of the Equality Challenge Unit, Higher Education Academy, and the Leadership Foundation for Higher Education in 2018. Codiroli-Mcmaster (2021) recent report identified the “BAME” awarding gaps as a difference of “9.9 percentage points in the academic year 2019/20” (Codiroli-Mcmaster, 2021, p. 2). The Office for Students (2021) reports that when “BAME” is disaggregated into racialized categories, the largest gap is revealed to be between Black and white students at 18.3 percentage points, nearly double that of the reported “BAME” gap. Here, we see that the award gap for students racialized as Black is nearly double that of the BAME award gap. This highlights one issue in the aggregation of non-white racialized categories into one homogenous group. The aggregation of BAME or BME in this way does not provide a comprehensive analysis of the differences between and across individual racialized categorizations compared with the normative or majority racialized grouping of white.

Whilst both organizations recognize the progress in the sector that has been made by higher education institutions in narrowing the award gaps, they explain that some of the factors contributing to degree awarding gaps are structural and therefore, deep-rooted. For example, noting that entry qualification, subject of study, or age of students contribute to degree awarding gaps (Office for Students, 2021). In addition, they acknowledge that any progress to close awarding gaps cannot be attributed to any intervention or factor due to the multifaceted nature of the gaps, further exposing the wicked problem.

Addressing the degree award gap from various approaches became a high priority of HEIs nationwide after the Office for Students (2018) called for a new approach to regulate access and participation within higher education. The proposed new approach requires the elimination of the gaps in access, progression, and degree outcomes within a 5-year period (2020–2025), particularly those based on ethnicity, disability, and POLAR quintiles as found within their analysis. As such, over 112 HEIs have made a formal commitment to close racialized degree awarding gaps between BAME and white students through the formulation of measurable targets within institutional access and participation plans (Office for Students, 2018). A consequence of the implementation of this policy by OfS is its greater awareness of the racialized degree awarding gaps within HEIs and across the higher education sector.

However, awareness is one thing; effective action is another. The degree award gap is a long-standing issue that has recently become a strategic priority for universities, because of the targets set by OfS in 2018 and the associated work of the mandatory Access and Participation Plans (APPs). Despite the recent heightened awareness of degree award gaps, there has been little progress at the sector level in addressing degree award gaps. Dr. Natasha Codiroli-McMaster explains in the “ethnicity awarding gaps 2019/20” report for Advance HE that “results from regression analyses showed that, overall, awarding gaps decreased, but persisted, even after controlling for qualifiers' individual and institutional characteristics” (Codiroli-Mcmaster, 2021, p. 2). This lack of progress in closing the degree award gap between racially minoritized students and white students was highlighted by the Office for Students (2022) report. The report showed that whilst the award of “good honours degrees” (classified as 1:1 or 2:1) were rising for BAME students, even more white students than in previous years, were awarded a 1:1 degree classification degree. As such, improvements in the rate of awarding good degrees to racially minoritized students have increased, but more white students than ever before are achieving the top classification of the degree.

The ongoing work and associated targets set by OfS and Advance HE mean that higher education institutions are actively developing plans to address the racialized degree award gaps in their institutions. However, a measurement approach to addressing degree award gaps only tells part of the story of inequitable outcomes across different racialized groups. The inadequacy posed by current measurement methods adopted in the sector is evidenced by the identification of the “unexplained gaps” (Codiroli-Mcmaster, 2021; Office for Students, 2022) that continue to be consistently persistent and pervasive with each new academic year. Unexplained gaps occur when the measured degree award gap between racialized groups cannot be explained by quantitative methods of analysis.

We need approaches to addressing degree award gaps that are exploratory and solutions focused. Solutions are inherently social, which reframe the outcome of 'problem solving' into 'a shared understanding of possible solutions' (Conklin, 2009, p. 18):

“shared understanding means that the stakeholders understand each other's positions well enough to have intelligent dialogue about their different interpretations of the problem, and to exercise collective intelligence about how to solve it.”

Austen et al. (2017) explain that wicked problems should be addressed by first looking at the surrounding infrastructure. At a macro level, this could be a better understanding of the social, economic, and political structures that operate and through which inequitable outcomes, such as racialized degree award gaps, are happening. At a meso level, we propose that trying to gain a shared understanding of the problem and working toward solving it (notwithstanding our position that degree award gaps are a wicked problem) means a better understanding of the experiences of stakeholders and their positions relative to each other and the system facilitating inequitable outcomes for those navigating it.

It is not enough to simply measure degree awarding gaps or the measurable factors associated with the gaps. We need to adopt an exploratory, solutions-focused approach to better understand the system and the racialized experiences of those in it. Therefore, we suggest a mixed methods approach (rather than quantitatively measuring degree award gaps only) to be adopted by the sector. In doing so, we can adopt a solutions-focused approach to better understand and therefore be better positioned to mitigate the risks and issues associated with racialized degree awarding gaps for students who experience systemic racism. The evidence of persistent, pervasive, and ongoing degree award gaps between white students and “BAME” or BME students over the last 25 years (Codiroli-Mcmaster, 2021) suggests that it is racially minoritized students who experience systemic racism. Systemic racism “is said to occur when racially unequal opportunities and outcomes are inbuilt or intrinsic to the operation of a society's structures. Simply put, systemic racism refers to the processes and outcomes of racial inequality and inequity in life opportunities and treatment” (Banaji et al., 2021).

## Current approach to addressing racialized degree award gaps

‘No person has a single, simplistic unitary identity' (Rollock and Gillborn, 2011, p. 4). Therefore, in seeking to develop ways to address degree awarding gaps, although our focus centers on racialized experiences, it is important to recognize this in approaches of measurement and analysis.

As such, the measurement of degree awarding gaps should be undertaken across different social locations of intersections. Bauer et al. (2021) explain that intersectionality is a theoretical framework rooted in the premise that human experience is jointly shaped by multiple social positions (e.g., race and gender) and cannot be adequately understood by considering social positions independently.

The current approach to the measurement of degree award gaps in the sector identifies social positions independently, as different independent variables. The relationships between the different variables are measured using multivariate data relationships. This is a flawed approach to better understanding degree award gaps, as it assumes that regardless of, for example, racialized categorization, variables inter-relate in the same way. This is simply not true—how gender and race inter-relate for a Black woman is different from how gender and race inter-relates for a white woman like my co-author. They are both unique sites of analysis. It is this notion that as different categorizations of variables create not just multiple, but different social locations that are not acknowledged in adopting the current regression method used by HEIs to analyse degree award gaps. Not only do variables, inter-relate differently, but the sites of analysis also associated with them are unique. The sites of analysis are not an aggregation of multiple characteristics; a common misconception of intersectionality. Crenshaw (1989) explains that these intersections, the unique sites of analysis, are reconstitutive and not additive. Consequently, these unique sites of analysis cannot be measured as a static and fixed social locations, but they can be better understood through exploration of lived experience over time.

The current methods of analyzing racialized degree award gap analysis using multivariate data relationships to measure gaps, simply tell us how distinct groups of students categorized into different racialized groupings experience the system. The methods use degree awards as a proxy for achievement or attainment. The reframing of achievement or attainment in this context is in recognition of the multiplicity of factors that contribute to student success and how institutional structures and discrimination can affect this (Loke, 2022); Again, recognizing that it is the award designated by the system, rather than an individual's attainment or achievement that is being measured.

This issue of discrimination wielded by the system impacting different groups of students with intersecting characteristics is beginning to be recognized in the sector, through the use of language and the lens used for the analysis of degree award gaps. For example, Advance HE launched its “ethnicity awards gaps in UK higher education” report in 2019/20, 10 years after the Equality challenge unit and higher education academy report “Improving the degree attainment of Black and minority ethnic students”'. Here, this is a clear development of language from attainment to awarding, which has since been adopted by the sector in recognition of the multiplicity of factors that contribute to student success, and how institutional structures and discrimination can affect this (Loke, 2022); This positioning moves away from the deficit ascribed to students from different racialized groups to one that recognizes that discrimination within the system. In the context of race, this is “systemic racism”.

As we have previously noted in the paper, with social justice work, just because the problem is complex, it does not mean that we accept the status quo. In the spirit of disruption to the status quo and a solution, action-based approach, we offer points of consideration and evaluation in relation to, what we deem, an enhanced method to the existing practice of measuring degree award gaps in the sector. We suggest that an intersectional lens be used in analyzing the lived experience of students, at the unique social locations of interaction, through narratives collected via qualitative data. These narratives of students who are not racialized as white are framed as counter-stories because they are counter to the normative, centralized positioning of social locations of whiteness. Additionally, we propose that quantitative data analysis of degree award gaps adopt dynamic centring to recognize the central positioning of whiteness in making comparisons of analysis.

Ruth Frankenberg explains that whiteness is embodied in three different ways:

1) Structural advantage of race privilege, which we see most clearly in terms of inequitable outcomes.
2) As a standpoint or the place from which people look at themselves and others in society—meaning proximity in relation to self.
3) In the form of practices, procedures, and policies that are unnamed and unmarked, and unnoticed by white people as having any relation to whiteness.

The evidence of degree award gaps between students racially categorized as white and students who are racially minoritized is the embodiment of whiteness in the system. The orientation of social locations and unique sites of analysis of students not racialized as white is always in relative proximity to white students (the dominant group); hence, the gap is framed as the “BAME or BME awarding gap”.

The current approach to addressing racialized degree award gaps is steered by targets outlined in access and participation plans (APPs). These are plans set by higher education providers to improve equality of opportunity for underrepresented groups to access, succeed in, and progress from higher education (Office for Students, 2022). It is within these APPs that progression targets are set, and therefore, activity to address degree award gaps is articulated. In February 2022, the OfS issued “refreshed priorities” to the sector, for providers to amend existing APPs and submit new plans for 2024/25. Given the student lifecycle emphasis on APPs, the activity associated with this work is often more prevalent at the institution level, where the resources required for a more comprehensive level of analysis are sparse. The sector approach to the measurement of award gaps is to measure and evaluate the gaps, relative to the measurable targets set by the OfS. Of course, in higher education policy, it is the case that “what gets measured, gets done.” As such, the sector is busy working on activities outlined in their APPs to close degree award gaps.

In trying to better understand factors impacting degree awards (and therefore what actions to take to address degree award gaps), higher education institutions adopt statistical testing to examine relationships between factors and the impacts on degree awards. Correlation (relationships between the variables) and/or regression analysis (strength of the relationships and whether they impact the outcome being measured i.e., degree award) are adopted to explain the relationship between variables, such as prior attainment, socio-economic background, sex, ethnicity, mode of study or mission group, and subject area, and their importance, relative to the degree award gap. Indeed, this analysis of correlation and/or regression is then used by the sector to make statistical inferences relating to the variables that can be predictors of degree award gaps. For example, Broecke and Nicholls (2007) seminal report, Ethnicity and Degree Attainment explains that even after controlling for most factors (i.e., variables) that we would expect to have an impact on attainment, being from a minority ethnic community (except the “Other Black”, “Mixed”, and “Other” groups) is still statistically significant in explaining final attainment, although the gap has been significantly reduced” (Broecke and Nicholls, 2007, p. 3).

“Statistically significant” in the context of the method adopted by the sector simply means the award gaps between students racially categorized as “BAME” (except for “Other Black”, “Mixed” and “Other” groups) compared with white students are not down to chance. This is affirmed by more recent research such as, Universities UK and National Union of Students (2019), Williams et al. (2019), and Ugiagbe-Green et al. (2021). Although statistical inference issues drawn from regression analyses conducted are important in the context of degree awarding gaps, there are other issues associated with the method of measurement that contributes to the limitations of existing methods used to analyse the degree award gaps.

This actual measurement of degree awarding gaps using quantitative methods is flawed in numerous ways. The methods to calculate degree awards across the sector and at the institutional level are hugely variable in themselves, as highlighted by Professor Burgess (2007) report “Beyond the honors degree classification”. This means that the criteria upon which a degree award is made differs across different institutions in the sector, as such, the basis on which the degree award outcome being measured across different higher education institutions across the sector differs. Additionally, the aggregation of students racialized as not white into categories such as “BAME” and “BME” is often an attempt to provide a more even group sizing to allow valid statistical comparison between racially minoritized students and those who are racialized as white. This is because some racially minoritized groups are under-represented (i.e., lower in number/proportion) in higher education, which poses an issue for statistical techniques to be applied for analysis. Consequently, a tactic employed by universities is to create two comparable groups (white and BAME/BME) for statistical testing, from which statistical inferences about the factors (variables) contributing to the degree award gaps can then be drawn. For example, “*T*-tests” are used to determine if there is a significant difference between the averages of two groups and whether the difference is statistically significant. This mode of analysis frequently used by higher education institutions in the sector to analyse differences between different racialized groups of students, requires that the groups are of similar size (Kim and Park, 2019). However, a consequence of aggregating non-white groups of students into one group, i.e., BAME or BME, means that disparities across and within different categories of racialized groups, i.e., unique sites of analysis, are hidden and a headline, overarching narrative of comparative difference, relative to those sites of analysis, i.e., BAME or BME, compared with white students, is reported, as noted in the Background section of this paper.

There is also a problematic assumption in that these variables, e.g., ethnicity (used as a proxy for race), socio-economic background (e.g., index of multiple deprivation; IMD), mode of study (part-time/full-time), and disability (e.g., neurodiversity), were used to analyse and better understand what contributes to degree award gaps, and have an equal weighting of importance on the outcome being measured and analyzed. The current method assumes that variables across all categorizations interplay and inter-relate with each other in the same way. As such, techniques such as correlation coefficient (the degree to which the movement of two variables is correlated) are used to determine the statistical significance of the relationship between two variables. This is problematic in that the site of analysis with two variables that are the same will be different for different racialized groups. The categorization of variables of ethnicity (a proxy for race) and gender do not interplay or inter-relate in the same way. Therefore, to better understand the racialized experiences of, for example, a cis-gender woman student racialized as Black compared with a cis-gender woman student racialized as white, we must recognize that the intersections are unique. This should be reflected in our statistical modeling of inferential analysis.

Zuberi and Bonilla-Silva (2008, p. 178) propose that;

“Statistical models that present race as a cause are really statements of association between the racial classification and a predictor or explanatory variable across individuals in a population. To treat these models as causal or inferential is a form of racial reasoning.”

Race is not a determinant or causal factor of degree award. Whilst measurement and understanding of lived experience will help us better understand the factors leading to degree awarding gaps between white students and those not racialized as white, it will not solve the wicked problem of degree awarding gaps. We propose this, because in better understanding how different racialized students experience the system, this in and of itself does not address issues of structural racism.

Critically, it is proposed that this inherent instrumentalist, measurement, target-based approach to addressing the problem of degree award gaps in the sector is not useful in the pursuit of change. The focus is on “the success or otherwise of strategies to improve outcomes, where the university experience is reduced to metrics such as retention and grade point average” (Nichols and Stahl, 2019, p. 1257), which cannot be examined in isolation from individual or social factors. Therefore, a more integrated approach to exploring experience and not just a focus on the outcome is needed to better understanding degree award gaps.

## The unexplained gap

The need for a more integrated approach to addressing racialized degree award gaps is highlighted by the existence of the “unexplained gap” prevalent when adopting the current approach to the measurement of the award gap. The “unexplained” gap occurs when quantitative methods do not explain fully the factors influencing the degree award gap (Cramer, 2021, p. 2). Codiroli-Mcmaster (2021) from Advance HE explains that when controlling for institutional factors (mission group, subject area, part/time, or full-time) and individual factors (age, gender, disability status, socio-economic background, and prior attainment), there is still an unexplained gap between students who are racialized as white and students who are racialized in non-white categories. This unexplained gap, which has been reported in HESA data since 2002/03, is systemic racism/structural racism or bias Codiroli-Mcmaster (2021). Powell (2007) explains that structural racism operates as the macro level systems, social forces, institutions, ideologies, and processes that interact with one another to generate and reinforce inequities among racial and ethnic groups. Structural mechanisms do not require the actions or intent of individuals (Bonilla-Silva, 1997). Structural racism is a system of oppression that impacts adversely on specific racialized groups and cannot be measured by quantitative data alone.

Therefore, we suggest that in trying to better understand degree award gaps, a mixed methods approach that also incorporates qualitative data, to provide insights into the lived experiences of students, will help to explain the “unexplained” gap. Most importantly, in adopting this approach, an intersectional lens to better understand the unique sites of analysis that cannot be measured by quantitative measurement alone can be taken.

For example, the existing measurement of the gap between a white cis-gender woman and a Black cis-gender woman in the system measures the gap in exactly the same way. Variables of gender and ethnicity (used as a proxy of race) are used as part of the measurement to analyse the achievement of degree awards. However, what the measurement does not explain, for example, is that when specifically thinking about race, a white woman does not experience misogynoir, a social site of inequity where racism meets sexism (Bailey, 2021). This unique site of inequity associated with the experience of a woman racialized as Black within the system cannot be captured by measurement of gender and ethnicity (a proxy for race variables) measured in the same way.

## Developing the current approach through an intersectional lens

In acknowledging the limitation of the current approach in addressing degree awarding gaps, we propose that we must adopt an intersectional (Crenshaw, 1989) lens to better understand students' racialized experiences during their student journey to determine how we can address systemic racism. Arising from Critical Race Theory (CRT) and regarded as a key development from “traditional equity research” (Naylor et al., 2016), this “non-traditional” (Collins, 2000) approach of intersectionality has been utilized within disciplines exploring inequalities within education. Intersectionality recognizes that particular characteristics or identities “do not operate as distinct categories of experience but are lived conjointly” (Nichols and Stahl, 2019, p. 1256), providing unique sites of analysis. Within this approach, Crenshaw (1991) identifies the concept of “structural intersectionality” which “refers to how multiple social systems intersect to shape the experiences of, and sometimes oppress, individuals” (Museus and Griffin, 2011, p. 7). This we see within the “unexplained gap”, particularly within the identification of, and the relationship between the institutional and individual factors as previously mentioned.

Adopting an intersectional lens for analysis allows for unique groups to be identified and the marginal effects of that group can be better understood. To take a non-intersectional approach through isolating factors associated with degree award gaps, without consideration for the complexities of how different racialized groups of students (with different intersecting characteristics) experience the system in different ways, potentially raises further barriers, placing individuals at risk of further disadvantage. It is at these unique sites of inequity, experienced by specific racialized groups, that systemic racism is experienced and exposed. A racialized lens is needed to better understand the lived experience of all students within the system. This provides insights into how systemic racism operates, is experienced, and how it might be mitigated and addressed.

It is the importance of adopting this racialized lens approach, to better understand the “unexplained gap” that arises from adopting quantitative analysis only and to better understand the experience of students within the system, which highlights the importance of critical race theory (CRT) as a foundational theoretical underpinning within the context of analyzing racialized degree awarding gaps. CRT was developed in the 1980s by legal scholars in the US, Derrick Bell, Kimberlé Crenshaw, and Richard Delgado. Today, it is being applied across different disciplines in the UK. The theory centers on race as a social construct. The philosophy underpinning the theory is critical realism; acknowledging that experience and understanding of the world is culturally, socially, and historically situated. That our experience of the world is shaped by our interactions with social systems; political power, social organization, and language. This theory underpins the experience of different racialized groups of students within the system and is the lens through which experiences of different racialized groups of students need to be analyzed. In CRT, experiences are told as narratives. In the case of people who are racialized as non-white, these narratives are counter-narratives. Ladson-Billings and Tate (1995, p. 56) define counter-narratives as “naming one's own reality” or “voice” by critical race theorists through “parables, chronicles, stories, counter-stories, poetry, fiction and revisionist histories”. Counter-narratives are positioned as such, because of their (distanced) proximity to the experiences of people racialized as white within the system.

## Dynamic centring

In giving voice to students in these different social locations to provide counter-narratives of how they see the world, our enhanced approach to better understanding different sites of analysis allows us to interrogate what McCall (2005) refers to as “intra-categorical intersectionality”, or differences within groups, as well as “inter-categorical intersectionality”, or differences between groups, to further our understanding of racialized experiences at these social locations. In doing so, for example, we disaggregate the homogenized racialized framing of “BAME” and “BME” and explore insights within and between the racialized categories. Building upon the intersectional approach, dynamic centring recognizes the relationality across different variables and their intersections within and across, for example, different racialized groups. It is in adopting dynamic centring as an approach to interrogating different sites of analysis that allows, for example, misogynoir to be identified, highlighted, and explored in the data analysis.

Current analysis within the context of racialized degree award gaps of the experience of students and the associated degree award outcomes is done by comparing that of students racialized as white with students not racialized as white. Therefore, the context in which the understanding of racialized experiences and degree award gaps is gained is in comparison with the “white experience” or embodiment of whiteness as measured by degree awards to students racially categorized as white, the dominant racial group. Dynamic centring is important in this context, as it is rooted in relationality. That is, at its foundation is the notion that there are differences between and within different categories of analysis. In recognition, Collins et al. (2009, p. 594) explains;

“Using dynamic centring for multiple social groups with diverse configurations of race, ethnicity; sexuality, class, age, gender, ability and citizenship status should expand sociology knowledge even further. Continuing this ongoing process of dynamic centring should, over time, yield a more complex and robust understanding of … multiple sites of inequality whether, health, education, or law enforcement.”

The dynamic aspect of this process of centring recognizes the temporal nature of positionality and proximity. The reality for the sector is that in the context of racialized degree awarding gaps, the positionality of students not racialized as white compared with students who are racialized as white (within the system), in terms of degree outcome, has remained largely static for at least two decades. At no stage since 2002/03 has the degree award gap inversed in favor of “BAME” students.

So, whilst the temporal nature of positionality in the system does not currently play out for students not racialized as whiteat the sector level (because their position has remained static for over 20 years), what dynamic centring does allow for are both inter-categorical (between) and intra-categorical (within) intersectionality. These approaches provide deeper insights into the experiences of different groups within the racialized category of “Black” between “Black British,” “Black African,” Black Caribbean, and Black Other relative to that of white. Of course, it can also be used to explore differences across groups too. For example, across gender (female, male, non-binary or transgender) and ethnicity (white) and disability (neurodiversity), relative to the dominant group.

It is also important to note, that policy changes, e.g., OfS 2019 targets and 2022 refreshed priorities to 112 higher education providers on closing “BAME” degree award gaps by 2024-25 (Office for Students, 2022), mean that the positionality/proximity of racialized students within the system may change. As such, dynamic centring is also an important methodological approach to evaluate any changes within this context.

## Regression analysis

There are different ways in which factors that are deemed important to how different students experience the higher education system, using degree award outcomes as a proxy for achievement, may be identified. In terms of quantitative data analysis, the most commonly used method to analyse degree award gaps is regression analysis. Regression analysis is a way of mathematically determining which of those factors, i.e., variables, have an impact on degree awards. It answers the questions: Which factors matter most? Which can we ignore? How do those factors interact with each other? And, most importantly, how certain are we about all these factors? (Gallo, 2015). In regression analysis, the factors identified in a study are referred to as variables. As such, regression analyses aim to determine the relationships and influence of explanatory (independent) variables on the thing, e.g., outcome (in this case, degree awards), being researched. The higher education sector uses regression analysis to better understand the factors that influence/impact degree awarding gaps, as well as to determine our certainty that we know all the factors. It can be suggested that the “unexplained gap” exists because the individual level (student) and institutional level (higher education institution) variables used to explain the gap do not identify all the factors that contribute to the racialized degree award gap.

Importantly, the association between different variables should not be interpreted as meaning that “innate” or “cultural differences among students not racially categorized as white', is causing relationships between degree awards (the outcome). Instead, what the regression analysis tells us is that those individuals racialized as not white may be subjected to different treatment, opportunities, and exposure to structural, institutional, and interpersonal racism than other individuals, in this context (see Jones, 2001, Zuberi, 2001, and Zuberi and Bonilla-Silva, 2008). The relationship between factors (variables) such as gender, race, and socio-economic background provides sites of analysis at which race categories intersect with other variables to form unique social locations.

Although other variables will influence degree award gaps, to better understand the contributing factors influencing the outcome, a racialized lens of analysis is required, which is our rationale for the use of CRT. The empirical value of a theory lies formidably in the balance between its explanatory power and predictive power (Shmueli, 2010). CRT can be used to predict inequities within our social structures. Although, CRT tends to have a higher degree of explanatory rather than predictive power than alternative propositional theories in racial studies (Jogie, 2022).

Gillborn et al. (2017, p. 171) explain this system takes an existing inequity (the lower attainment of previous generations of Black students) and uses it to predict a future where such inequity is normal. As such, CRT can and does predict the existence of racialized degree award gaps. This is a critical historical and contextual position because it acknowledges that it is the experience of these students within the system that leads to the relationships that are measured by marginal effects of the system, rather than issues/deficits inherent within the students themselves. In the context of degree award gaps, we propose that CRT is used as a lens of analysis. However, the tenets of the theory, also add weight to our framing of degree award gaps as a wicked problem, principally due to what Derrick Bell refers to as the “permanence of racism”.

This racialized lens is furthered through the development of QuantCrit. Garcia et al. (2018), describe QuantCrit as “critical quantitative intersectionality.” Covarrubias and Vélez (2013) developed a QuantCrit framework and suggested that numbers are contextualized and do not “speak for themselves'; that quantitative analysis is grounded in experiential knowledge and standpoints; that research, like the tenets of CRT, is designed to advance social justice; and those transdisciplinary approaches are necessary (Covarrubias and Vélez, 2013). This is reflected in the position proposed by Zuberi and Bonilla-Silva (2008), who urge all researchers to employ racial statistics for racial justice. That is, in seeking to address the inequity of degree awards across different racialized groups, researchers must understand and acknowledge the historical and cultural contexts in which the statistical models are applied to explain and predict racialized degree award gaps.

## A conceptual framework

This methodological discussion of this paper is within the context of framing the racialized degree award gaps as persistent, pervasive, in flux, and ultimately, a wicked problem. As such, we believe that degree award gaps are impossible to solve. The system in which different racialized groups of students' experience is not fixed. The system and how students experience it is relational to your racial categorization and proximity to whiteness. This can be temporal (although evidence of the existence of the “BAME” or “BME” award gap spanning at least 25 years suggests not) and is certainly messy and non-linear. This underpins the need for an approach that adopts, dynamic centring in analyzing how different groups of students experience the system in relation to that of the dominant group; students racialized as white. The method also allows for deeper exploratory analysis within and across different racialized groups. Critically, you cannot deracialise discourse or the impact of racialized experiences when analyzing degree awarding gaps. It is not prior attainment, sex, gender, socio-economic background, mission group, mode of study, and so on, that is central to understanding degree award gaps; it is race.

Cramer (2021, p. 11) states, “for the past 10 years, only 0.5% per year has been shaved off the undergraduate degree award gap” (AdvanceHE, 2020). Yet, in the same period, there have been extensive reviews and reports that have detailed why the unexplained award gap exists and hundreds of recommendations on how universities can tackle it (Berry and Loke, 2011; Alexander and Arday, 2015; Amos and Doku, 2019). Yet, Loke (2022) suggests that at this rate of change, it will be in 2070–2071 when the white-BAME awarding gap will close, and 2085–2086 when the white-Black awarding gap closes. However, the refreshed targets set by OfS to the 112 higher education institutions to close the “BAME” award gap by 2024–25 suggest that perhaps the degree awarding gap will close at a more rapid rate.

Whilst it is our view that adopting a mixed methods approach in the way outlined in the paper will not solve the degree awarding gap problem itself, it will help us to better understand the experiences of different racialized groups of students within the system. To better understand lived experience, Cramer (2021) explains that “engaging with the lived experiences and views of minority ethnic students and staff is fundamentally important for developing potential key solutions. In the context of identifying areas that might help speed up gap closure, balancing these with quantitative approaches.” (p. 14).

As well as adopting a mixed method approach, through an intersectional lens, we propose that it is important to contextualize racialized experiences. We propose that quantitative data should be analyzed using dynamic centring (which essentially measures proximity to the dominant group) and that this data, in the context of degree award gap measurement, should not be deracialized. As such, we recommend that the approach to qualitative data analysis is underpinned by CRT. It is through the racialized lens of CRT that counter-narratives explain the experiences of racially minoritized students, who do not have close proximity to the dominant group and who have intersecting characteristics, giving rise to unique sites of inequity are best understood. The inequitable outcomes relating to degree awards ascribed to students who are not racialized as white are a proxy of the experience and achievement as awarded by higher education institutions in the system.

Hence, it is our view that whilst approaches and methods can be adopted to better understand degree award gaps and the associated issues relating to them at points in time, the complexities, the temporal shifts (which we may now see due to the 2025 target set by the OfS), and the pervasive and permanent nature of systemic racism, means that racialized degree award gaps, will remain a wicked problem.

This paper is partly a call to action to the higher education sector to prove us wrong!

## Conclusion

This paper provides a conceptual contribution to the emerging field of QuantCrit, but we are hopeful that the paper will also impact on policy of analyzing degree award gaps in Universities. Our critical discussion of quantitative methods of analysis contributes to the discourse on how quantitative methods and CRT can be combined (Garcia et al., 2018). However, we also endeavor to provide methodological guidance that may be necessary for those seeking to move these premises into research practice, an issue identified by Sablan (2019).

As demonstrated, there is a significant problem with the way in which awarding gaps are measured, which is by the statistical inference that race (by proxy, ethnicity) is a causal variable of degree awarding gaps. The outcomes of regression analysis undertaken to establish the likelihood of achievement of a “good degree” by separate groups of racialized students are not a predictor of the attainment of a “good degree”, but a proxy for achievement and how different racialized students have experienced the system. Furthermore, the performance indicator, i.e., outcome of “degree awarding gaps”, is a measure that simply tells us what the measured gaps in relation to the degree award assigned to students categorized into homogenized ethnicity categories (BAME and BME), compared with students who are racialized as white. Degree award gaps as measured simply indicates how different students experience the system using degree awards as a proxy for this as an outcome of the award. Further analysis of racialized experiences through narratives provides insights and a lens through which to better understand students' experience of the system and a means of identifying avenues for change. For students racially minoritized, these counter-narratives provide insights into experiences such as misogynoir.

In conclusion, one of the features of degree award gaps as a wicked problem is the framing of the problem itself. The complexity associated with the factors impacting student experience and cultural and historical legacies that are embedded in systemic policies, procedures, activities relating to admissions, assessment, curricula, and many other formal and informal activities and spaces during the student lifecycle means that racialized degree awarding gaps will forever persist, because it is the system and not the student that is the root problem.

## Author contributions

All authors listed have made a substantial, direct, and intellectual contribution to the work and approved it for publication.

## Conflict of interest

The authors declare that the research was conducted in the absence of any commercial or financial relationships that could be construed as a potential conflict of interest.

## Publisher's note

All claims expressed in this article are solely those of the authors and do not necessarily represent those of their affiliated organizations, or those of the publisher, the editors and the reviewers. Any product that may be evaluated in this article, or claim that may be made by its manufacturer, is not guaranteed or endorsed by the publisher.
